# Diet, nutrition, and hormone therapy for prostate cancer: a systematic review with implications for future interventions

**DOI:** 10.1093/jncics/pkag014

**Published:** 2026-02-17

**Authors:** Isabella Pahulu, Matthew Calumpit, Paul Tominez, Jonathan J Shih, Sasha Ebrahimi, Nicole V Deville, Raynald Samoa, Tannaz Moin, Mina S Sedrak, Luca F Valle, Michael Steinberg, Amar U Kishan, Patricia A Ganz, Kekoa Taparra

**Affiliations:** Department of Epidemiology and Population Health, Stanford University, Stanford, CA, United States; Department of Biophysics and Biochemistry, University of Pennsylvania, Philadelphia, PA, United States; Department of Surgery, Tripler Army Medical Center, Honolulu, HI, United States; School of Medicine, University of California, San Francisco, San Francisco, CA, United States; Department of Radiation Oncology, David Geffen School of Medicine, University of California Los Angeles, Los Angeles, CA, United States; Department of Epidemiology and Biostatistics, University of Nevada, Las Vegas, Las Vegas, NV, United States; Department of Diabetes, Endocrinology, and Metabolism, City of Hope, Duarte, CA, United States; Department of Medicine, Division of Endocrinology, Diabetes, and Metabolism, David Geffen School of Medicine, University of California Los Angeles, Los Angeles, CA, United States; Endocrinology Service, Greater Los Angeles Veterans Affairs Healthcare System, Los Angeles, CA, United States; Department of Medicine, Division of Hematology/Oncology, David Geffen School of Medicine, University of California Los Angeles, Los Angeles, CA, United States; Department of Radiation Oncology, David Geffen School of Medicine, University of California Los Angeles, Los Angeles, CA, United States; Radiation Oncology Service, Greater Los Angeles Veterans Affairs Healthcare System, Los Angeles, CA, United States; Department of Radiation Oncology, David Geffen School of Medicine, University of California Los Angeles, Los Angeles, CA, United States; Department of Radiation Oncology, David Geffen School of Medicine, University of California Los Angeles, Los Angeles, CA, United States; Department of Health Policy and Management, Fielding School of Public Health, University of California Los Angeles, Los Angeles, CA, United States; Department of Radiation Oncology, David Geffen School of Medicine, University of California Los Angeles, Los Angeles, CA, United States; Department of Health Policy and Management, Fielding School of Public Health, University of California Los Angeles, Los Angeles, CA, United States

**Keywords:** prostate cancer, androgen deprivation therapy, hormone therapy, diet, nutrition, systematic review, ROB2, creatine, Mediterranean diet, low-carbohydrate diet, vitamins, supplements

## Abstract

**Background:**

Given excellent prostate cancer outcomes, comorbidity management is critical to survivorship. While hormone therapy or androgen deprivation therapy (ADT) is a mainstay of treatment, they can negatively impact quality of life and survivorship through cardiovascular, sexual, and metabolic effects. ADT-induced metabolic syndrome causes impaired glucose tolerance, muscle mass loss, and weight gain. This systematic review examined recent randomized clinical trials (RCTs) investigating the impact of diet and weight management strategies on mitigating ADT-related adverse effects.

**Methods:**

A systematic review of RCTs (2015-2025) was performed using PubMed/Embase following Preferred Reporting Items for Systematic Reviews and Meta-Analyses (PRISMA) guidelines. To identify how diet and weight management impacts ADT symptoms, search terms included: “prostate cancer,” “diet,” “nutrition,” “glucagon-like peptide-1 receptor agonists” (GLP-1RA), and “ADT.” Risk of Bias 2 (ROB2) and Grading of Recommendations Assessment, Development, and Evaluation (GRADE) tools evaluated RCT quality.

**Results:**

Of 2799 publications, 16 met inclusion/exclusion criteria (range, 23-96 patients/RCT). No RCTs had a high risk of bias or evaluated GLP-1RA. Outcomes included metabolic labs, body composition, and quality of life. Mediterranean and low-carbohydrate diets with exercise reduced cardiovascular and metabolic risk factors, with variable durability. Creatine trended toward increasing lean muscle mass. Multidisciplinary care and community involvement improved accountability and outcome durability.

**Conclusions:**

This comprehensive review of diet and ADT in prostate cancer identified nutritional interventions that were safe, feasible, and may be recommended as part of prostate cancer treatment and survivorship. Future RCTs should evaluate optimal diet duration, longer follow-up, multidisciplinary patient support, and novel anti-metabolic therapies like GLP-1RA.

## Introduction

Prostate cancer is the most common non-cutaneous malignancy in men, contributing to nearly one-in-three cases of male cancers, with a prevalence of over 3.5 million survivors across the United States (US) in 2025.^1^^,^^2^ Given men with prostate cancer have among the highest survival rates of all cancers, proper treatment and diagnosis of non-cancer comorbidity burden is fundamental for cancer survivorship.^1^ Diabetes and obesity are prevalent in the general population, with an estimated global disease burden of 590 million (US: 39 million) and 2 billion (US: 170 million) adults, respectively.^3-5^ Among men with prostate cancer, nearly one-third are diagnosed with type 2 diabetes mellitus (T2DM) or obesity.^6^ T2DM and obesity can dysregulate inflammatory, neoplastic, and hormonal pathways, which in turn may promote tumor progression and metastasis.^7^^,^^8^ Although advanced age is the most influential risk factor for prostate cancer, diabetes and obesity can impact prostate cancer risk and survivorship.^9-14^

Androgen deprivation therapy (ADT) is a mainstay of treatment for aggressive prostate cancers. However ADT can negatively impact metabolic, cardiovascular, sexual, and cognitive function, and quality of life.^15^ Patients on long-term ADT have an increased risk of not only obesity and diabetes, but also coronary heart disease, osteoporosis, dementia, and sexual dysfunction.^1^^,^^16^ Patients receiving ADT have a higher incidence of diabetes, and those with preexisting diabetes can experience worsening diabetic control.^16^ Thus, lifestyle optimizations, including diet and nutritional supplements, may be important considerations to enhance survivorship outcomes.^17-19^

Recent studies demonstrated that certain diets (eg, high Healthy Eating Index diets and fish oil capsule supplementations) can lower the risk of prostate cancer reclassification to higher risk disease.^20^^,^^21^ Prior systematic reviews have evaluated dietary modification in the setting of men with prostate cancer treated with ADT, but these studies did not include nutritional supplements in the search parameters, did not focus on diet exclusively (eg, included exercise and behavioral modifications), and investigated outcomes beyond ADT side effects (eg, prostate specific antigen).^22^^,^^23^ There is an unmet need for a comprehensive and contemporary literature review of dietary intervention RCTs that relate to improving the adverse effect profiles of ADT in prostate cancer treatment.

In conjunction with diet optimization, there is growing interest in pharmaceutical management of metabolic syndromes for patients with prostate cancer. For example, arm K of the STAMPEDE trial compared standard of care with metformin versus without metformin among non-diabetic men with high-risk locally advanced or metastatic hormone-sensitive prostate cancer undergoing ADT.^24^ The trial demonstrated that the adverse side effects of ADT, including weight gain and dyslipidemia, were significantly reduced with metformin. Additionally, glucagon-like peptide 1 receptor agonists (GLP-1RAs) are a type of incretin mimetic-based therapy increasingly prescribed to manage multiple aspects of metabolic syndrome comorbidities, including T2DM and obesity.^3^^,^^25^ Emerging evidence suggests GLP-1RAs confer an estimated 20-50% risk reduction in prostate cancer diagnosis.^26-28^ Due to their effectiveness with weight management and glucose homeostasis maintenance, GLP-1RAs are a compelling target for treatment to help with prostate cancer survivorship.^3^ Thus, the purpose of this systematic review was to examine the contemporary RCT literature for diet, nutritional supplement, and/or pharmaceutical-based interventions that may mitigate ADT-related adverse events.

## Methods

### Publication search strategy

A systematic search of PubMed and Embase was conducted in August 2025, adhering to the Preferred Reporting Items for Systematic Reviews and Meta-Analyses (PRISMA) guidelines.^29^ To capture any trials relevant to dietary or nutritional interventions in prostate cancer including possibly GLP-1RA treatments, the following *a priori* search term strategy was used to identify the initial set of articles for evaluation: (“prostate cancer” OR “prostatic neoplasms”) AND (“nutrition” OR “diet” OR “nutritional interventions” OR “glucagon-like peptide-1 receptor agonists” OR “GLP-1” OR “GLP1” OR “semaglutide” OR “liraglutide” OR “exenatide” OR “tirzepatide”) AND (“androgen deprivation therapy” OR “ADT” OR “hormone therapy” OR “androgen suppression” OR “hormonal treatment” OR “lupron” OR “leuprolide” OR “leuprorelin” OR “bicalutamide” OR “enzalutamide” OR “apalutamide” OR “darolutamide”).

### Study inclusion/exclusion criteria

We limited our search to English-language studies from the past 10 years (2015-2025) to capture modern ADT utilization practices and GLP-1RA treatments. We included studies focused on (1) men with prostate cancer undergoing ADT, (2) diet/nutrition intervention, and (3) study outcomes pertaining to ADT-induced side effects (eg, lab values, metabolic syndrome, fatigue, vasomotor symptoms). We excluded studies that were not human RCTs (eg, reviews, retrospective studies, observational studies).

### Data extraction and quality assessment

Two authors (IP, KT) performed the initial data extractions using search criteria and then performed individual study title, abstract, and full-text article screens using the Covidence platform.^30^ For each included article, the following data were collected: author, year, country, patient sample size, patient population, nutrition intervention, ADT side effects addressed, study arms, outcome measures, and results. Three authors reviewed and confirmed the extracted data (MC, PT, JS). Two authors (IP, MC) independently assessed the study quality using the Risk of Bias 2 (ROB2) tool for RCTs using signaling questions to determine each study’s risk of bias.^31^^,^^32^ Quality assessment conflicts were resolved by a third reviewer (KT). The authors (IP, MC, KT) evaluated the certainty of the evidence for clinically meaningful outcomes following the Grading of Recommendations Assessment, Development, and Evaluation (GRADE) guidelines using the GRADEpro software.^33^^,^^34^

## Results

### Randomized clinical trials and quality assessment

#### Included RCTs and study characteristics

**Figure 1.**
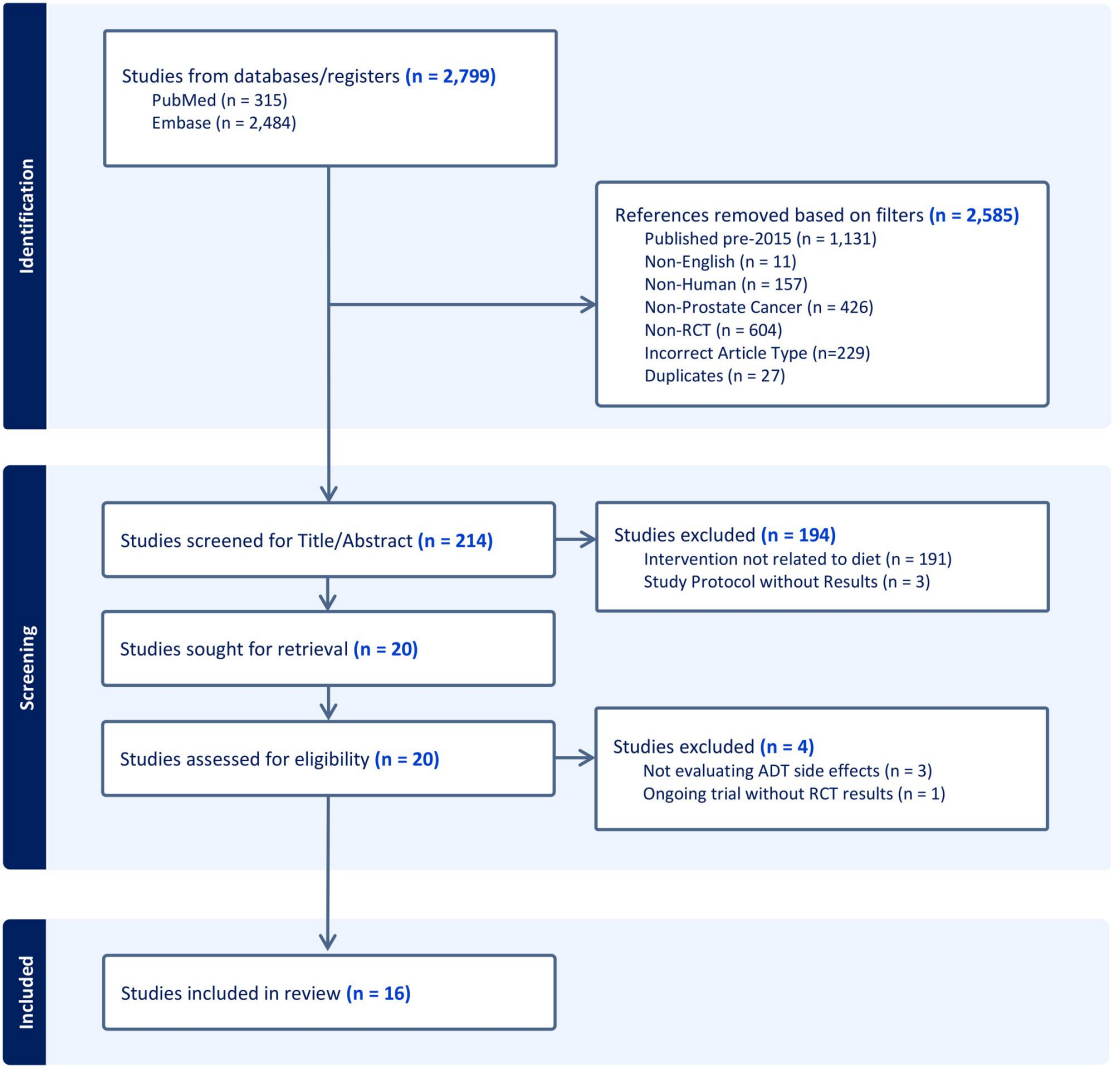
PRISMA file for selection of studies to include in systematic review.

**Figure 2.**
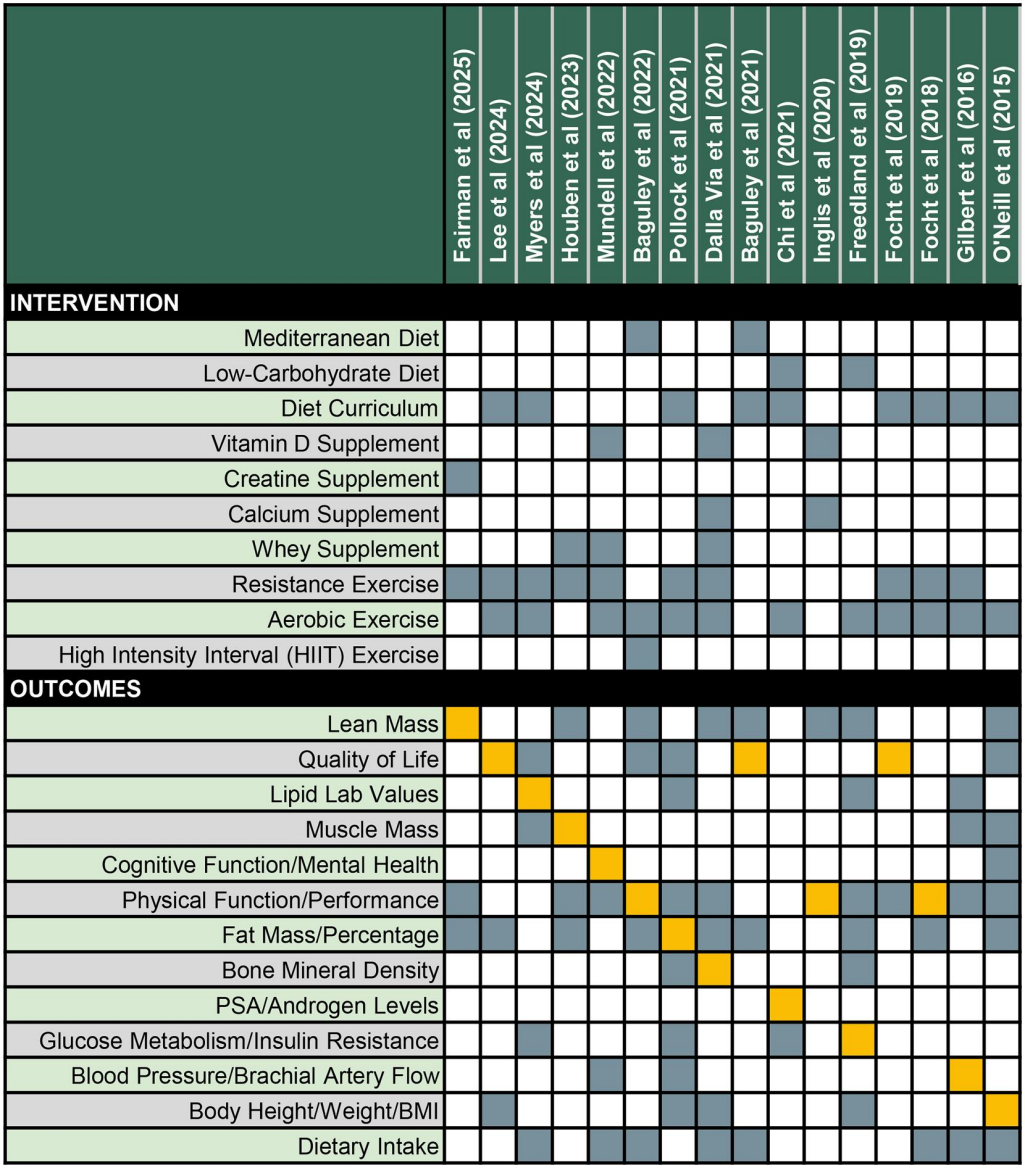
Heat Map of Interventions and Outcomes based on the 16 included RCTs. For outcomes, the orange squares represent primary study outcomes and the blue/grey squares represent non-primary outcomes.

Among 2799 screened, 16 publications met the inclusion criteria (Table 1; Figure 1). The analytic sample included 13 unique RCTs with sample sizes ranging between 23-96 patients per RCT. Two publications^35^^,^^36^ were explicitly preplanned analyses of a RCT outcome, while 3 publications^37-39^ were described as either exploratory, hypothesis generating, or an ancillary analysis of an original trial. Follow up was on average 6 months (range, 3-12 months). Figure 2 displays the intervention components and outcomes of each RCT. These RCTs evaluated diet (*n* = 4), diet curriculum (*n* = 9), supplements (*n* = 5), and exercise (*n* = 14). The impact of these interventions was studied on a diverse set of outcomes, including physical function and performance (*n* = 12), body composition (fat mass *n* = 10, lean mass *n* = 8, muscle mass *n* = 4), dietary intake (*n* = 8), and health-related quality of life (*n* = 7). All participants had either started or were planning to start ADT for at least 3 months. Fourteen RCTs combined exercise with either dietary or supplement interventions.

**Table 1.** Summary of 16 randomized clinical trials in men with prostate cancer on ADT and the effect of nutritional interventions.

Publication (*n*)	Prostate cancer population	Study arms	Nutritional intervention	Outcomes	Results
**Fairman, 2025** ***Western Australia*** (*n* = 30)	No bone metastases No synchronous cancer No medications known to alter body composition Not participating in regular resistance training Not taking creatine in the past 6 months	Double-blind, randomized, placebo-controlled RCT Placebo vs creatine Both groups underwent resistance exercise training Stratified by time on ADT (≤6 vs >6 months) and age (≤65 vs >65 years)	Intervention arm received 20 g/day of creatine monohydrate for 5 days (divided into 4 equal doses throughout the day) starting immediately after randomization, then received 5 g/day for the remainder of the 12-week intervention	Lean mass (whole body and appendicular)* Fat mass (whole body and trunk, body fat percentage) Muscle strength (chest press, leg press, seated row) Physical function (chair rise, timed up and go, 400-meter walk)	Trend towards increased lean mass with resistance training (MD = 0.3) Significant increase in fat mass only within placebo group (not intervention), but no between-group differences Significant within-group improvement in muscle strength across the 12-week period for both study arms Resistance exercise with or without creatine may facilitate benefits in strength and lean mass
**Myers, 2024** ***United States*** (*n* = 38)	Men ≥21 years ADT within 3 months of study enrollment Reinitiation of ADT after prior ADT holiday English or Spanish speaking Consistently reachable by telephone during the study Ability to travel to the institution for baseline, 6-month, and 12-month follow up	Randomized control trial Intervention arm: Individualized aerobic and resistance exercise program, in-depth nutrition curriculum focusing on heart health and blood sugar control, and education regarding how ADT works and accompanying adverse effects Control arm: No systematic education on nutrition and physical activity	Individualized nutritional curriculum based on the National Cholesterol Education Program, Adult Treatment Panel III Therapeutic Lifestyle Changes guidelines, and the American Institute of Cancer Research’s healthy eating guidelines Focus on portion control and increase in intake of non-starchy vegetables, plant-based proteins, fish, and fat-free or low-fat dairy Emphasis in minimizing charred foods and trans fats Frequent check ins by study nurses to assess nutrition and activity goals Assessment of metabolic, cardiovascular, and health-related quality of life measures at the 6-month mark	Low-density lipoprotein (LDL)* Total lipid levels (total cholesterol, HDL, and triglycerides) Fasting blood glucose Hemoglobin A1c Health-related quality of life assessment (Short Form Health Survey version 2, Expanded Prostate Cancer Index Composite Short Form) Nutrient Intake Diet quality (Alternative Healthy Eating Index)	No between group differences for LDL and other cardiovascular outcomes Significant decrease in HDL within intervention group only (d = −0.39) Global improvement in metabolic profile with frequent nutritional counseling Overall, showed feasibility of a nutritional and exercise educational intervention
**Lee, 2024** ***South Korea*** (*n* = 46)	ADT ≥6 months at the time of enrollment Abnormality of ≥1 of 5 metabolic syndrome components (fasting blood sugar, abdominal circumference, blood pressure, fasting triglyceride level, fasting HDL cholesterol) Understood spoken and written Korean without cognitive impairment Access to a smartphone Exclusion criteria: non-prostate cancers; recent surgery or chemotherapy (<3 months prior); “yes” response to ≥ 1 Physical Activity Readiness Questionnaire item; cardiovascular disease or other comorbidities; other research enrollment; changes to blood pressure, cholesterol or blood sugar medications within 3 months before study; difficulty with typical daily activities	Single-blind, single-center randomized waitlist control trial Each participant was assigned to an experimental or a waitlist control group in the order of enrollment so that all participants underwent the health coaching program at some point	Nurse-led mobile-based health coaching program focused on individualized diet, exercise, and strategies to manage ADT-induced side effects Four-week program conducted 1-on-1 via Zoom meetings followed by an 8-week maintenance program conducted via KakaoTalk	Changes in the Lifestyle Evaluation Tool for Metabolic Syndrome Patients score* Changes in the 5 metabolic syndrome components Body composition (waist circumference, skeletal muscle mass, total lean mass, appendicular lean mass, total fat mass, and fat percentage) Changes in the Expanded Prostate Cancer Index Composite score	Improvement in lifestyle scores (F = 38.49) and improvements in urinary irritative and obstructive symptoms (F = 7.01) Patients had improvements in fasting blood sugar (F = 3.98) and reduced abdominal circumference (F = 4.09) Reduction in body weight (F = 10.71) and BMI (F = 10.49) Lifestyle changes should be implemented consistently for at least 3 months
**Houben, 2023** ***Netherlands*** (*n* = 96)	ADT ≥6 months Exclusion criteria: Unable to participate in exercise training; comorbidities severely limiting physical activity; high risk for pathological fractures due to bone metastases; life expectancy <1 year; lactose intolerance or whey protein allergy; cognitive disorders or severe emotional instability; inability to speak, understand, or read Dutch	Multicenter partly randomized-controlled trial, comparing 2 intervention groups with a separately recruited control group Intervention groups: Randomly allocated in a double-blinded fashion to resistance exercise training with either protein supplementation or placebo supplementation Control group received usual care	Exercise group consumed either a protein or placebo supplement directly after every exercise session and each night before sleep Protein supplement contained 31 g whey protein, 13 g carbohydrate, and 1 g fat Placebo supplement contained 1 g protein, 12 g carbohydrate, and 0.4 g fat Patients were instructed to keep a food diary	Skeletal muscle mass* Body composition (total lean mass, appendicular lean mass, total fat mass, and fat percentage) Muscle strength (leg press, leg extension) Physical performance (timed up and go, 30-second chair stand test, stair climb test) Aerobic capacity (maximal workload, peak oxygen uptake, peak respiratory exchange ratio)	Muscle mass and strength increased in both exercise+protein and exercise+placebo, compared to control group Total fat mass and fat percentage increased in exercise+protein (η²ₚ = 0.127) and control group (η²ₚ = 0.145) but not in the exercise+placebo Increase in 1-repetition maximum and aerobic capacity (eg, VO_2_ peak, workload max) with both exercise groups compared to the control group Protein supplementation is not required but should be investigated for all patients who are not nutritionally optimized at baseline
**Mundell, 2022** ***Australia*** (*n* = 70) *secondary analysis*	Men aged 50-85 years with prostate cancer and currently on ADT for 12 weeks or greater Exclusion criteria: Inability to communicate in English; current smoker; body weight >159 kg; pre-existing disorder or medication affecting bone, calcium, or vitamin D metabolism; current protein supplementation; participation in resistance or impact training within the past 3 months; contraindications to exercise testing	A 12-month, single-blinded, 2-arm randomized controlled trial Intervention arm: 12-mo exercise training program with daily nutritional supplement Control group: Ongoing care from their physician and a daily 1000U vitamin D supplement	Intervention group participants were supplemented with 25 g whey protein concentrate mix and 1000 IU vitamin D prior to each exercise session and prior to breakfast on non-training days	Cognitive function (Trail making test, Rey Auditory Verbal Learning Test, Digit span forwards and backwards test, National Adult Reading Test, CogState Brief Battery)* Blood pressure Physical activity and diet Depression and anxiety (Depression and Anxiety Stress Scale)	No significant between-group differences in cognitive function at 6- and 12-month follow ups Nutrient supplementation arm showed within group improvement in verbal recall, verbal working memory, and the CogState working memory score at 12 months Both arms showed within group improvement in immediate recall, verbal learning, visuomotor speed, and task switching at 12 months Multicomponent exercise training alone or with nutritional support may not be a viable therapeutic intervention to improve cognitive function in men treated with ADT for prostate cancer who are cognitively intact at baseline
**Baguley, 2022** ***Australia*** (*n* = 23)	Men ≥18 years or older Non-smoker or had quit smoking for at least 3 months Diagnosis of prostate cancer and treated with ADT for at least 3 months BMI between 18.5-34.9 kg/m^2^ Exclusion criteria: Use of supplements other than a single multivitamin (unless medically indicated); musculoskeletal, neurological, respiratory, metabolic, or cardiovascular conditions limiting safe participation; current infection; bone metastases	A single-blinded 2-arm randomized controlled trial Intervention arm: 20 weeks of MED-diet and starting at week 12, started an 8-week HIIT program Patients also received dietary consultations every 2 weeks Control arm received usual care	Mediterranean-style dietary pattern meeting the following targets: ○ > 10% total energy from saturated fat ○ 2 servings/day fruit ○ 5 servings/day vegetables ○ 30 g/day fiber ○ Reduce or eliminate red/processed meats ○ 3 servings/week fish ○ 2 servings/day dairy ○ 1 servings/day nuts and seeds ○ ≤ 2 units of alcohol/week Aerobic high intensity interval training (HIIT): 4 x 4 min 85-95% heart rate peak, 3x week.	Cardiorespiratory fitness* Body composition (fat mass, lean mass, and body fat mass) Cancer-related fatigue and quality of life measures (Functional Assessment of Chronic Illness Therapy: Fatigue (FACIT-F), Functional Assessment of Chronic Illness Therapy: General (FACIT-G), Medical Outcomes Study 36-Item Short-Form Health Survey) Dietary intake	Mediterranean diet plus HIIT significantly improved cardiorespiratory fitness at 20 weeks compared to usual care Mediterranean diet plus HIIT reduced body weight at 20 weeks compared to usual care, with no change in lean body mass Mediterranean diet plus HIIT significantly improves vitality, mental health composite, prostate cancer specific quality of life, and cancer-related fatigue at 20 weeks compared to usual care
**Baguley, 2021** ***Australia*** (*n* = 23)	Men ≥18 years or older Non-smoker or had quit smoking for at least 3 months Diagnosis of prostate cancer and treated with ADT for at least 3 months BMI between 18.5-34.9 kg/m^2^ Exclusion criteria: Use of supplements other than a single multivitamin (unless medically indicated); musculoskeletal, neurological, respiratory, metabolic, or cardiovascular conditions limiting safe participation; current infection; bone metastases	Two-arm randomized controlled trial Intervention arm: face-to-face nutrition consultations with a dietitian every 2 weeks for 12 weeks Control arm received usual care	Mediterranean-style dietary pattern meeting the following targets: ○ > 10% total energy from saturated fat ○ 2 servings/day fruit ○ 5 servings/day vegetables ○ 30 g/day fiber ○ Reduce or eliminate red/processed meats ○ 3 servings/week fish ○ 2 servings/day dairy ○ 1 servings/day nuts and seeds ○ ≤ 2 units of alcohol/week	Cancer-related fatigue and quality of life measures (Functional Assessment of Chronic Illness Therapy: Fatigue (FACIT-F), Functional Assessment of Chronic Illness Therapy: General (FACIT-G), Medical Outcomes Study 36-Item Short-Form Health Survey)* Dietary intake (Wollongong Dietary Inventory) Body composition (fat mass, lean mass, and body fat percentage) Inflammatory markers	Mediterranean diet improved cancer related fatigue and quality of life at 12 weeks compared to usual care Mediterranean diet reduced total body mass, lean body mass, and IL-8 at 12 weeks compared to usual care Mediterranean-diet is safe and feasible and has potential to improve cancer-related fatigue and quality of life in overweight men treated with ADT
**Pollock, 2021** ***United States*** (*n* = 48)	Men with prostate cancer who had started ADT < 6 months prior to enrollment with ADT planned for at least 12 months following enrollment Eastern Cooperative Oncology Group performance status of 0 to 2 Ability to exercise on one’s own Concurrent radiation therapy was allowed Exclusion criteria: Receipt of chemotherapy within 12 months prior to enrollment and/or presence of permanent pacemaker or implantable medical device except for artificial joint prostheses or venous filters	Phase II single institution randomized study Intervention arm: STAND Clinic Control arm received usual care, but referral to available services were allowed and at the discretion of the patient’s oncologist	Participation in the STAND clinic had visits every month for 12 months Patients received individualized in-person counseling with a registered dietitian, licensed exercise trainer, or a palliative care specialist on a rotating basis for a total of 4 visits with each service Individual diets were reviewed and personalized recommendations were provided by a study dietitian	Percent body fat* Metabolic profile (insulin resistance, hemoglobin A1c, fasting lipids, waist circumference, BMI, and blood pressure) Bone health (serum vitamin D level and bone density of lumbar spine, femoral neck, and total hip on DEXA scan) Quality of life (PHQ-9, Attention Functional Index, Lee Fatigue Scale, International Prostate Symptom Score, Expanded Prostate Cancer Index-Composite Short-Form, Hot Flash Related Daily Interference Scale, 12-Item Short Form Survey) Physical activity	No significant differences in primary and secondary outcomes between in-person counseling intervention and usual care groups at 12 months Majority of STAND clinic visits were completed, emphasizing the feasibility Trend towards some improvements in metabolic profile but none were statistically significant Study confounded by 39% of control group being referred for intervention services
**Dalla Via, 2021** ***Australia*** (*n* = 60)	Men age 50-85 years with histologically diagnosed prostate cancer and treated with ADT for longer than 12 weeks Exclusion criteria: Disorders affecting bone or calcium/vitamin D metabolism; use of bone-active medications other than ADT; prior protein, calcium, or vitamin D supplementation within 3 months; regular weight-bearing exercise within 3 months; current smoker; weight >159 kg; absolute contraindications to exercise testing	Two-arm, 12-month randomized controlled trial Intervention arm: exercise + nutrient supplementation Control arm received usual care	Nutrient supplement consisted of a 25 g whey protein-, 1200 mg calcium-, and 1000 IU vitamin D-enriched drink combined with a 1000 IU vitamin D tablet	Hip and spine areal bone mineral density* Volumetric bone mineral density Body composition (lean mass and fat mass) Muscle strength (leg press, chest press, seated row 3-repetition maximum) Physical function (30-s sit-to-stand test, timed up and go, 4-square step test, Berg Balance Scale, 4-m usual walk, 400-m walk) Intervention adherence Anthropometry (BMI) Physical activity (Community Healthy Activities Model Programme for Seniors questionnaire) Diet	No significant difference between groups on bone or body composition outcomes at 12 months Improvement in leg muscle strength and dynamic mobility in exercise+supplement group versus control Per protocol analysis demonstrated improved lean body mass and femoral neck bone mineral density Exercise training combined with multi-nutrient supplementation did not improve adverse musculoskeletal consequences of ADT, though results confounded by modest intervention adherence
**Chi, 2021** ***United States*** (*n* = 29) *secondary analysis*	Men with prostate cancer about to begin hormonal therapy with an anticipated duration of at least 6 months and had a BMI of at least 24 kg/m^2^ Exclusion criteria: Medication-controlled diabetes; use of medications affecting insulin metabolism; current low-carbohydrate diet; vegetarian or vegan diet; hemoglobin A1c >7% at baseline	Unblinded, randomized controlled trial Intervention arm: patients received coaching from a study dietitian to reduce carbohydrate intake to less than 20 g/day and walk at least 30 minutes/day for 5 days a week Control arm received usual care	Low-carbohydrate diet	Androgen levels (eg, androsterone sulfate) and metabolic markers (ketogenesis, glycolysis, and amino acid metabolism) measured using metabolomic profiling* at baseline, 3 months, and 6 months Prostate specific antigen, glucose, high sensitivity C-reactive protein	Low-carbohydrate diet mitigated ADT-induced reductions in ketones and acyl-carnitines, and decreased lactic acid, alanine, and S-adenosyl methionine compared to usual care Low-carbohydrate diet disrupted correlations between androgens and glucose metabolism Low carbohydrate-diet did not affect androgen suppression, cholesterol, insulin, or high-sensitivity C-reactive protein
**Inglis, 2021** ***United States*** (*n* = 59) *secondary analysis*	≥ 60 years old No bone metastases ADT for ≥6 months with an additional 6 months planned Low vitamin D levels (<32 ng/mL) Total serum calcium (≤10.5 mg/dL) No contraindications for fitness testing Exclusion criteria: Adequate vitamin D levels; hypercalcemia; osteoporosis; stage IV kidney disease; myocardial infarction within the past year	Phase II RCT Arm 1 received weekly high-dose weekly vitamin D for 24 weeks Arm 2 received a weekly vitamin D placebo alongside a daily multivitamin containing 600 IU/day vitamin D + 1010 mg/day calcium	High dose vitamin D_3_ (50 000 IU/week) + daily multivitamin containing 600 IU/day vitamin D + 1010 mg/day calcium	Phase angle (bioelectrical impedance analysis)* Lean mass (bioelectrical impedance analysis) Physical function (handgrip test, knee extension test, short physical performance battery [SPPB] based on: standing balance test, walking speed for a timed 4-meter walk, repeated sit-to-stand or chair stand test)	High-dose vitamin D_3_ arm had significantly wider phase angle values at weeks 12 (MD = −0.461) and 24 (MD = −0.506), indicating healthier muscle cells Physical function and lean mass did not differ between arms and did not decline over the course of the study
**Freedland, 2019** ***United States*** (*n* = 40)	Men with prostate cancer about to begin hormonal therapy with an anticipated duration of at least 6 months and had a BMI of at least 24 kg/m^2^ Exclusion criteria: Symptomatic metastatic disease; medication-controlled diabetes; use of medications affecting insulin metabolism; current low-carbohydrate diet; vegetarian or vegan diet; baseline hemoglobin A1c >7%	Unblinded, randomized controlled trial Intervention arm: patients received coaching from a study dietitian to reduce carbohydrate intake to less than 20 g/day and walk at least 30 minutes/day for 5 days a week Control arm received usual care	Low-carbohydrate diet	Insulin resistance measured by homeostatic model assessment (HOMA)* Glucose metabolism Anthropometric measures Lipid metabolism and inflammatory measures Body composition and exercise measures Adverse events	Low carbohydrate diet and walking improved insulin resistance at 3 months compared to usual care, but was not significant at 6 months potentially due to low enrollment) Low carbohydrate diet improved weight, A1c, HDL, and triglycerides at 3 months compared to usual care; only weight loss and HDL remained significant at 6 months Low carbohydrate diet significantly preserved total body bone mineral count and reduced fat mass, lean mass, and percent body fat compared to usual care
**Focht, 2019** ***United States*** (*n* = 32) *secondary analysis*	Histologically defined diagnosis of prostate cancer Current ADT with a planned course of at least 3 months of continuous therapy Less than 60 minutes of structured exercise participation per week during the past 6 months Lack of poorly controlled medical conditions that preclude safe participation in exercise Consent to participate from the treating oncologist and primary care physician	Single-blind, 2-arm randomized controlled pilot trial Intervention arm: group-mediated cognitive behavioral counseling involving exercise and dietary guidance Control arm received usual care	Basic nutrition counseling, addressing contemporary topics in nutrition and cancer, personalized guidance towards adopting changes in dietary intake (diet rich in whole grains, vegetables and fruits; limited consumption of processed high-fat, low-nutrient dense foods; reduced intake of red and processed meats)	Self-efficacy (The Exercise Self-Efficacy Scale, Multidimensional Self-Efficacy for Exercise Scale, Mobility-Related Self-Efficacy Scale)* Satisfaction with Physical Function scale Satisfaction with Appearance scale Mobility performance and exercise participation (400-m walk test, Godin Leisure-Time Exercise Questionnaire)	Group mediated cognitive behavioral counseling with diet and exercise showed improvements in exercise (d = 0.66), coping (d = 0.63), and scheduling self-efficacy (d = 0.62), but no difference in mobility-related self-efficacy (d = 0.01) compared to usual care Satisfaction with physical function (d = 1.33) was increased in the outcome group compared to usual care Better social cognitive outcomes were associated with superior mobility performance and exercise participation
**Focht, 2018** ***United States*** (*n* = 32)	Histologically defined diagnosis of prostate cancer Current ADT with a planned course of at least 3 months of continuous therapy Less than 60 minutes of structured exercise participation per week during the past 6 months Lack of poorly controlled medical conditions that preclude safe participation in exercise Consent to participate from the treating oncologist and primary care physician	Single-blind, 2-arm randomized controlled pilot trial Intervention arm: group-mediated cognitive behavioral counseling involving exercise and dietary guidance Control arm received usual care	Basic nutrition counseling, addressing contemporary topics in nutrition and cancer, personalized guidance towards adopting changes in dietary intake (diet rich in whole grains, vegetables and fruits; limited consumption of processed high-fat, low-nutrient dense foods; reduced intake of red and processed meats)	Mobility performance (400-m walk test)* Mobility performance (stair climb) Muscular strength (one-repetition maximum) Body composition (body fat mass) Leisure-Time Exercise Questionnaire Dietary Health Questionnaire	Clinically meaningful improvements in mobility performance by 400-m walk test (d = 0.72) and stair climb test (d = 0.47) in cognitive behavioral counseling group compared to usual care Improved total body weight (d = 0.88), body fat % (d = 0.78), fat mass (d = 0.82), lower body (d = 0.89) and upper body muscular strength (d = 0.86), and self-reported resistance exercise sessions (d = 1.83) in cognitive behavioral counseling group compared to usual care
**Gilbert, 2016** ***England*** (*n* = 50)	Men with prostate cancer Sedentary lifestyle (exercising <90 min/week at a moderate intensity) Receiving continuous ADT for a minimum of 6 months prior to recruitment, with planned long-term retention on ADT Exclusion criteria: Men with unstable angina, uncontrolled hypertension, recent myocardial infarction, pacemakers, painful or unstable bony metastases	Two-arm, parallel-group, single-blinded randomized controlled trial Intervention arm: supervised 12-week lifestyle intervention including exercise and dietary advice Control arm received usual care, but no restrictions placed on them in terms of exercise or dietary behaviors	Nutritional counseling focused on reduction in dietary fat, increased consumption of fruit and vegetables, increased fiber consumption, and decreased intake of refined carbohydrates and limiting alcohol intake to 1-2 units per day	Brachial artery flow mediated dilatation (endothelium-dependent arterial dilatation)* Glyceryl trinitrate (GTN)-mediated brachial artery dilatation Resting blood pressure Treadmill walk time Body mass and composition Lipid profile Biomarkers of disease status Physical activity and dietary behaviors	Slight improvement in endothelial function *(*d = 0.60) in lifestyle intervention group compared to usual care Improvement in skeletal muscle mass, treadmill walk time (d = 1.41), skeletal muscle mass, flow mediated dilatation peak, exercise behavior (d = 0.57), and sex-hormone-binding globulin at 12 weeks in lifestyle intervention group compared to usual care In the acute period, diet and exercise may have a benefit in reducing cardiovascular risk factors while patients are on ADT
**O’Neill, 2015** ***Ireland*** (*n* = 94)	Histologically proven adenocarcinoma of the prostate LHRH agonist therapy plan to initiate or continue ≥6 months of LHRH therapy Exclusion criteria: Comorbid conditions limiting physical activity; medical conditions requiring a restricted fruit/vegetable diet; history of insulin-dependent diabetes; treatment with steroid hormones; prior treatment for another cancer; life expectancy <2 years	Two-arm parallel-group randomized controlled trial Intervention arm: home-based dietary and exercise counseling Control arm received usual care but received the full intervention at the end of the study	Patient’s diet was tailored to encourage them to meet these guidelines: ○ ≥5 servings of vegetables and fruits/day ○ 30-35% of total energy from fat and <10% energy from saturated fat/day ○ 10% of energy from polyunsaturated fat/day ○ Limited consumption of processed meats ○ 25-35g of fiber/day ○ ≤28 units/week of alcohol ○ Limit high salt/sugar foods	Body composition (weight, BMI, fat mass, lean muscle mass, waist to hip ratio, mid-upper arm muscle area)* Fatigue Severity Scale Quality of life (Functional Assessment of Cancer Therapy-Prostate) Functional capacity using the 6-min walk test Perceived Stress Scale Dietary intake	The diet and physical activity intervention group had a significant reduction in adverse body composition changes (weight, BMI, waist to hip ratio, waist and hip circumference, fat mass %, fat mass) associated with ADT compared to usual care No differences in lean body mass and mid-upper arm muscle between intervention and control groups Improvement in functional well-being, decreased emery consumption, and decreased total fat and saturated fat intake in interventional compared to usual care

Asterisk (*) defines the primary outcome.

#### Quality assessment

**Figure 3.**
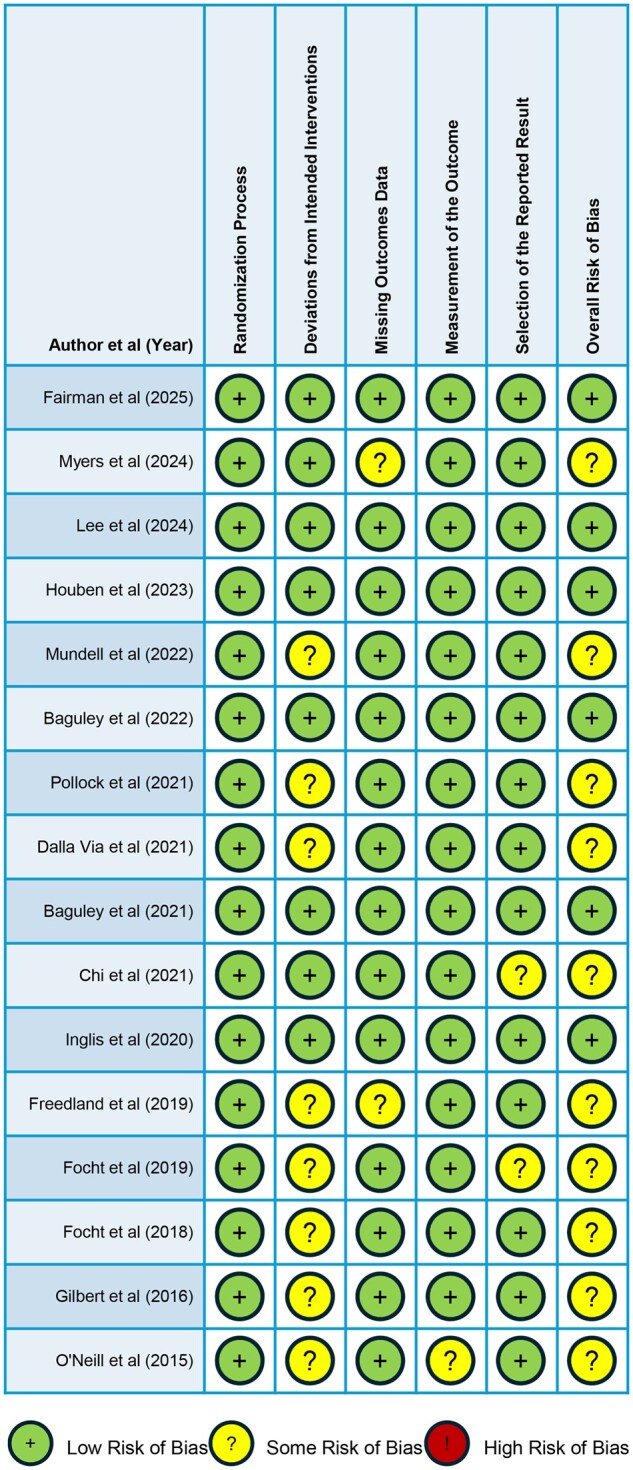
Risk of Bias 2 (ROB2) tool assessment of randomized clinical trial quality.

The ROB2 tool was used to assess RCT quality (Figure 3). No study had “high overall risk of bias.” However, 11 studies had “some overall risk of bias,” which was often attributed to deviations from an intended intervention.^17^^,^^35^^,^^37^^,^^40-44^ Among these, some interventions, including exercise, were not double-blinded, thus making it challenging to assess whether the study’s primary outcome measurement was biased by patient knowledge of the intervention.^44^ Multiple studies reported moderate adherence to exercise or diet interventions (eg, interventions including whey protein/calcium/vitamin D-enriched drinks, low-carbohydrate diet, and Mediterranean diet).^35^^,^^42^ Two RCTs had “some overall risk of bias” given missing outcome data, with patient data missing after randomization due to the lack of follow-up, adverse health events, patient withdrawal from the study, or progressive disease.^17^^,^^40^^,^^45^ Since 2 RCTs were secondary or post-hoc analyses without prior planning, they were classified as having “some overall risk of bias” given the potential of bias in the selection of the reported result.^38^^,^^39^

Evidence certainty was evaluated using GRADE appraisal (Table S1), with moderate certainty (diet intervention: weight/BMI, lean mass, fatigue, overall quality of life; supplement intervention: weight/BMI, lean mass), low certainty (diet intervention: low-density lipoprotein/high-density lipoprotein, vasomotor symptoms), and very low certainty (diet interventions: hemoglobin A1c/insulin resistance). The combination of diet (including low-carbohydrate diet or Mediterranean diet), exercise, and counseling most consistently improved fatigue, weight/BMI, lean body mass, and general quality of life metrics (Table S1).

### Nutrition and supplementation

#### Impact of diet on ADT side effects

An Australian RCT (Baguley, 2022) evaluated the effects of a Mediterranean diet with tailored counseling and a high-intensity interval training (HIIT) program on cardiorespiratory fitness, cancer-related fatigue, quality of life, and change in dietary intake in men with prostate cancer receiving ADT.^36^ Participants were on the Mediterranean diet for 20 weeks, which consisted of cruciferous vegetables, tomato, fruits, calcium/vitamin D-containing foods, polyunsaturated, monounsaturated, and omega-3/6 fatty acids.^46^ Participants concurrently received tailored dietary advice and an educational pamphlet to support at-home nutrition from an Accredited Practicing Dietitian every 2 weeks for 20 weeks. At week 12 of tailored dietary advice, participants initiated HIIT sessions in weeks 12-20. HIIT significantly improved cardiorespiratory fitness, and the Mediterranean diet with HIIT resulted in significant body weight reductions, compared to the usual care control. A limitation to this study was that patients were not stratified by BMI at randomization (intervention group mean BMI 27.4 kg/m^2^ versus control group mean BMI 30.6 kg/m^2^) and BMI was not accounted for in the analysis.

Two publications evaluated ADT side effects with a dietary regimen that improved metabolic outcomes (Table 1).^17^^,^^39^ The Carbohydrate and Prostate Study 1 (CAPS1) RCT evaluated whether a low-carbohydrate diet paired with daily walking impacted insulin resistance in men, with BMI ≥24, initiating ADT for prostate cancer at 6 months.^17^ The intervention arm (*n* = 19 patients) received weekly (months 1-3) and biweekly (months 4-6) individual coaching to reduce carbohydrate consumption to ≤20 grams/day and to walk ≥30 minutes/day, while the control group (*n* = 21 patients) maintained baseline dietary and exercise patterns. Although at 6 months the intervention did not result in significant improvements in insulin sensitivity (Homeostatic Model Assessment of Insulin Resistance), the study found improved secondary outcomes with the intervention group, including anthropometric measures (weight: −9.3 kg versus +1.3 kg; BMI: −9.0 kg/m^2^ versus +1.4 kg/m^2^), lipid metabolism (high-density lipoprotein: +36.3 versus +9.3), and body composition (fat body mass: −16.2 g versus +11.0 g; percent body fat: −8.4% versus +10.4%). The authors discussed the limitation of their small sample size, which impacted the power of their study. They proposed methods to reduce barriers to participation, such as delayed intervention and home visits for data collection.

Subsequently, Chi et al. analyzed the serum metabolome of 29 CAPS1 trial participants to understand the mechanistic pathways associated with low-carbohydrate diets.^39^ They identified that low-carbohydrate diets significantly improved metabolic health, including weight gain, insulin resistance, hemoglobin A1c, and triglycerides. One caveat was that the authors could not determine the individual impact of the daily walking compared to the low-carbohydrate diet, which provides directions for future studies.

One RCT evaluated dietary modifications in men with prostate cancer who underwent a 6-month nurse-led nutrition curriculum, in which nurses helped patients improve their food choices, prevent weight gain, maintain muscle mass via portion control, and provided a tailored diet and exercise plan.^40^ There were no significant between-group differences for the primary outcome of low-density-lipoprotein (LDL) levels. Significant within-group differences for high-density-lipoprotein (HDL; mean difference −0.4) and hemoglobin A1c (mean difference −0.2) were observed. Moreover, the study showed low attrition rates and high participant satisfaction.

#### Impact of nutritional supplements on ADT side effects

Five RCTs evaluated nutritional supplements including creatine, whey protein, and vitamin supplements.^35^^,^^38^^,^^42^^,^^47^^,^^48^ One exploratory analysis evaluated a high-dose vitamin D supplement (600 IU/day + 50 000 IU/week vitamin D + 1010 mg/day calcium) compared to a placebo control (600 IU/day + 1010 mg/day calcium + vitamin D placebo), which yielded a significant improvement in phase angle (indicating healthier muscle cells).^38^ While the primary study of Inglis et al. did not fit this systematic review inclusion criteria, notably, the study found that vitamin D daily dosage of 800 IU/day was associated with a significant reduction in hip and femoral neck bone mineral density loss.^45^ The loss of bone mineral density was seen more prominently among the patients who had low vitamin D intake at baseline.

For the 4 other studies, none of the primary outcomes were significantly changed with nutritional supplementation including patient body composition, cognition, or bone health (Table 1). Interventions lasted from 12-20 weeks. In contrast to diet based RCTs, these supplement trials were able to leverage a placebo control. Creatine was studied due to its known role for mitigating the loss of lean muscle, physical strength, and physical function among men.^47^^,^^49^ Most supplementation was delivered concurrently with exercise (ie, resistance training).

Given these negative studies, there were multiple caveats to these trials that the authors considered. The authors discussed that the 12-week intervention may have been too short to generate significant lean muscle mass increases. The patients reported by Houben et al. in their whey supplementation study were already consuming enough protein in their diet, thus the authors recommended a future study that enrolls older patients with unoptimized nutritional status.^48^ Mundell et al. noted that in their protein and vitamin supplementation RCT, 10 participants in the intervention arm started chemotherapy, which could have its own negative impacts on their primary outcome of cognitive testing.^35^ Dalla Via et al. commented in their study on a host of body composition endpoints that intervention adherence (56% exercise and 77% nutritional supplement) may have impacted the effectiveness of their trial. Nonetheless, despite multiple negative trials, the authors highlighted that there were no adverse events noted in this study, which provides guidance for healthcare providers when asked about supplements in clinic.

### Additional components of diet RCT designs

Given the complexity of dietary interventions in a RCT setting, there were multiple studies that included specific interventions beyond just modifying diet and nutrition itself. These studies evaluated strategies for patient communication, multidisciplinary clinics, patient education, or exercise combinations. The following section reviews some components of these studies that were tested by the researchers, but not solely with regards to the dietary intervention itself.

#### Patient communication modes

Patient communications were used as an interventional supplement in several studies (Table 1). A 2024 study in South Korea evaluated a mobile-based nurse-led educational intervention for 12 weeks and encouraged a low-fat and low-carbohydrate diet.^50^ Participants received health coaching from nurses via weekly Zoom or phone calls and received an educational package (PowerPoint slides, exercise and nutrition diaries, exercise video files, TheraBand resistance bands, and a pedometer) to supplement this at-home intervention. Instead of measuring LDL, this study measured lifestyle scores to assess the health behaviors of patients with metabolic syndrome symptoms. This study found reductions in fasting blood sugar, abdominal circumference, weight, BMI, urinary irritation, and urinary obstructive symptoms—components that are critical for prostate cancer survivorship.

Another 2024 study evaluated the effects of a 6-month nurse-led nutrition curriculum focused on heart health and blood sugar control.^40^ While the results were not significant, all participants reported that regular communication with a nurse added structure/incentive to increase their activity, and expressed a desire for communications to continue beyond the scope of the study. This RCT underscored the positive impact of regular and motivational communication during ADT to improve patient quality of life and satisfaction with received care.

#### Multidisciplinary clinic approaches

One phase II study (Pollock, 2021) examined the effects of a multidisciplinary Supportive Therapy in Androgen Deprivation (STAND) Clinic that provided individualized in-person counseling with a registered dietitian, exercise trainer, and palliative care specialist to patients with prostate cancer undergoing ADT for ≥12 months (Table 1).^41^ Every patient at the STAND Clinic met with each specialist 4 times across the 12-month study period, and participants received individualized dietary recommendations after reviewing dietary habits. During follow-ups, patients received feedback based on self-reported changes in dietary habits.

Although this study integrated several survivorship components in an individualized manner, there were no significant differences between the study arms in metabolic and bone health or quality of life (Patient Health Questionnaire, Attention Functional Index, Lee Fatigue Scale, International Prostate Symptom Score, Expanded Prostate Cancer Index Composite Short Form, Hot Flash Related Daily Interference Scale, and 12-Item Short Form Survey) between the study arms. This may be due in part to low trial accrual and existing intervention crossover of the control group (39% of the control group were referred to similar services of the intervention group including dietitian or physical therapist consult). The authors also discussed that 12 months may not have been long enough to yield significant results and called for interventions with longer follow-up periods.

#### Education and behavioral modification

A study conducted by Focht et al. measured the primary outcome of mobility performance and secondary outcomes including muscle strength, body composition, self-reported resistance exercise, and diet intake due to education and group-mediated cognitive behavior counseling (Table 1).^37^^,^^51^ This 12-week intervention provided dietary education and personalized guidance to adopt positive dietary behavior changes. They also received small-group counseling sessions after their personalized exercise sessions twice a week. The authors reported clinically meaningful improvements in mobility performance, measured by a 400-meter walk test, and that patients maintained short-term behavior change resulting from the counseling intervention.

Focht et al. conducted an ancillary analysis of the same intervention, which was the only evaluated RCT to take a social cognitive approach to improve ADT-related side effects.^51^ This approach led to meaningful improvements in social cognitive outcomes such as self-efficacy, satisfaction with physical function and appearance, mobility performance, and exercise participation, which related to more favorable levels of exercise participation.

One study conducted in England (Gilbert, 2016) measured the change in endothelial function in sedentary men who planned to be on ADT long-term after an exercise and nutrition education intervention.^43^ Endothelial function was measured by flow-mediated dilatation of the brachial artery. Participants received 20-minute small-group healthy-eating seminars, every 2 weeks, and were also prescribed 3 exercise sessions per week for the 12-week intervention. The guidance given during the seminars included: consumption of 5 portions of fruits and vegetables/day, increased fiber consumption, reduction in dietary fat intake to approximately 25% of total energy intake, decreased intake of refined carbohydrates, and limiting alcohol consumption to 1-2 units/day. The authors found that improved endothelial function during the first 6 weeks of supervision was lost at the endpoint assessments by week 24.

A German study also measured the impact of written patient information materials on physical activity and dietary patterns.^52^ The study suggested that patients in this sample were interested in receiving written lifestyle change recommendations, as 90% reported that they would make use of the information. However, urologists estimated that a large majority of their patients rarely or never followed the guidance, which points to the larger environment around the patients. Authors agree that the information would be helpful, but more complex strategies for long-term maintenance are integral.

#### Exercise

While exercise was not included directly in the search terms of this systematic review, exercise was a core component of 14 of the RCT interventions that aimed to mitigate negative ADT side effects with dietary or supplement interventions (Figure 2). One study found that even short-term resistance exercises could mitigate negative side effects by increasing lean body mass.^47^ When combined with dietary interventions, many study authors commented on the challenges of disentangling the effects of nutrition and exercise. However, 1 study from the Netherlands found that resistance exercise training potentially reversed the negative side effects on muscle mass and function, and found that supervised small-group exercise sessions resulted in benefits in social connection and the ability to adapt the program to individual needs.^48^ The authors conclude that prostate cancer specialists should consider prescribing exercise to all patients starting ADT, including those with bone metastases who are not at high-risk for pathological fractures.

### Specific androgen deprivation side effect management

#### Metabolic dysregulation

Fourteen studies highlighted improvement of several metabolic ADT adverse side effects with dietary interventions, particularly as secondary RCT outcomes (Figure 2). ADT impacts muscle (sarcopenia) and bone (osteopenia) metabolism, which can have poor long-term quality of life consequences.^53^^,^^54^ Dalla Via et al. demonstrated that their 12-month combined exercise and nutritional supplement (whey protein, calcium, and vitamin-enriched drink with vitamin D tablet) improved areal bone mineral density of the femoral neck, even after adjustment for treatment covariables including radiation therapy. The same study demonstrated the improvement of lean muscle mass. Gilbert et al. likewise demonstrated an improvement in skeletal muscle mass with a nutrition and exercise intervention.^43^

#### Fatigue

Fatigue is a common side effect of ADT, thus several RCTs evaluated some component of fatigue through either the Functional Assessment of Chronic Illness Therapy-Fatigue (FACIT-F) (*n* = 2), Lee Fatigue Scale (*n* = 1), and the Fatigue Severity Scale (*n* = 1) (Table 1).^36^^,^^41^^,^^44^ One RCT specifically measured cancer-related fatigue after a 20-week Mediterranean diet and HIIT training program, and found that cancer related fatigue improved after 12 weeks of diet modifications alone.^36^ However, the improvement diminished at week 20 (after introducing HIIT at week 12).

To evaluate the impact of nutrition alone, Baguley et al. measured cancer related fatigue, total body mass, and quality of life after testing the Mediterranean diet without the exercise component.^46^ The authors reported that this dietary intervention significantly improved all 3 outcomes. The authors discussed despite a limited sample size, significant efforts were made to increase enrollment through methods such as involving peers and partners in the study design to improve retention and incorporating telehealth to increase accessibility.

Another study evaluated the impacts of a 6-month home-based exercise and dietary intervention on fatigue, quality of life, and stress.^44^ While it resulted in significant improvements in weight reduction, BMI, and percentage fat mass, it did not significantly impact fatigue, quality of life, or stress. The authors hypothesized that fatigue related to long-term use of ADT may be more difficult to mitigate by diet and exercise behavior change alone.

#### Vasomotor symptoms

Vasomotor symptoms including hot flashes, which are characterized by feelings of warmth, flushing, sweating, are also common side effects of ADT. Pollock et al. evaluated the impact that diet had on symptomatic relief of hot flashes due to ADT. The STAND clinic study included the Hot Flash Related Daily Interface Scale measurement.^41^ The increase from baseline in Hot Flash Interference Score was 15 points lower (100-point scale) in the STAND clinic intervention arm, compared with the usual care control arm. This signified a trend in improved hot flash symptoms with the multidisciplinary intervention arm. However, while this was not statistically significant, potentially due to the heterogeneity in both groups, this may be clinically significant for some patients as it approaches the study’s 16.6 minimally important difference threshold.^55^

ADT hot flashes may also specifically impact quality of life through negative impacts on sleep. Myers et al. noted that men in their study reported negative impacts on their sleeping patterns due to hot flashes.^40^ Hot flashes ranged from “not too bothersome” to “terrible” in the patient population of an RCT that conducted qualitative interviews.^40^ The diet modification alone ultimately did not influence hot flashes.

#### Sexual function

Nutrition and exercise have been evaluated as an intervention to mitigate negative sexual functioning and sexual bother side effects. Myers et al. was 1 study that found a statistically significant within-group difference in worsening sexual function (*Cohen’s d *= 0.56) and sexual bother (*Cohen’s d = *0.70) at 6 months, compared to baseline scores. This finding suggests that nutrition and exercise may delay worsening of sexual bother, which warrants further investigation.

### Adverse events

Generally, all RCTs in this systematic review showed diet and nutritional supplement interventions were safe and did not cause any major adverse events. However, there were some reports of minor adverse side effects. For example, there were some reports of mild GI bother as a result of interventions including the 25 gram whey protein, 1200 mg calcium, and 2000 IU vitamin D supplement.^35^^,^^42^ There were rare reports of fatigue, headaches, and constipation after a low carbohydrate diet with moderate walking.^17^ Other studies reported various minor musculoskeletal complaints that resulted in exercise modifications.^35^

## Discussion

Our systematic review identified 16 RCTs that tested nutrition and dietary interventions for men with prostate cancer undergoing ADT. For clinicians, this review highlights that nutrition interventions (including the low carbohydrate diet or Mediterranean diet) are generally safe and feasible as part of survivorship care for men with prostate cancer undergoing ADT, but require future prospective clinical trials to evaluate comparative efficacy. The addition of dietary optimization has the potential to mitigate metabolic side effects of ADT such as weight gain, fasting hyperglycemia, and insulin resistance. One study focusing on high-dose vitamin D supplementations showed some benefit to muscle health, but the 3 remaining trials focusing on dietary supplementation (eg, whey protein, creatine, and calcium) were negative for their primary outcomes. Nonetheless, there were no studies included in this review that demonstrated harm, albeit with small sample sizes overall in the included studies. This systematic review identified aspects of studies that were feasible, but also signals a call for action for further research to expand the potential impact of these dietary interventions and related metabolic optimization to improve outcomes for patients with prostate cancer treated with ADT.

### Metabolic risk reduction and longitudinal care

Nutrition plans and increased physical activity show promise in mitigating these ADT related effects especially when implemented and maintained over time. Further investigation into these effects is necessary as durable responses were more common with 6 months of intervention compared with assessments at 3 months post-intervention. These benefits also emphasize the challenge of sustaining metabolic improvements without ongoing support. The transient intervention benefits likely signal challenges with patient adherence to the investigated interventions following the conclusion of the RCT. Therefore, investments in clinical trials that assess interventions that are tolerable and self-sustaining, especially when supplemented by social support networks, are warranted.

Studies that leveraged multidisciplinary team members (eg, nurse-led lifestyle coaching) significantly improved fasting glucose and waist circumference. These multidisciplinary teams were effective in communicating to patients the importance of behavioral modifications to improve quality of life. These studies suggest that ADT related metabolic side effects can be modified through targeted lifestyle change when coordinated appropriately. However, sustained reversal of adverse metabolic changes requires effective long-term programming beyond the initial intervention period.

### Body composition and musculoskeletal health

**Figure 4.**
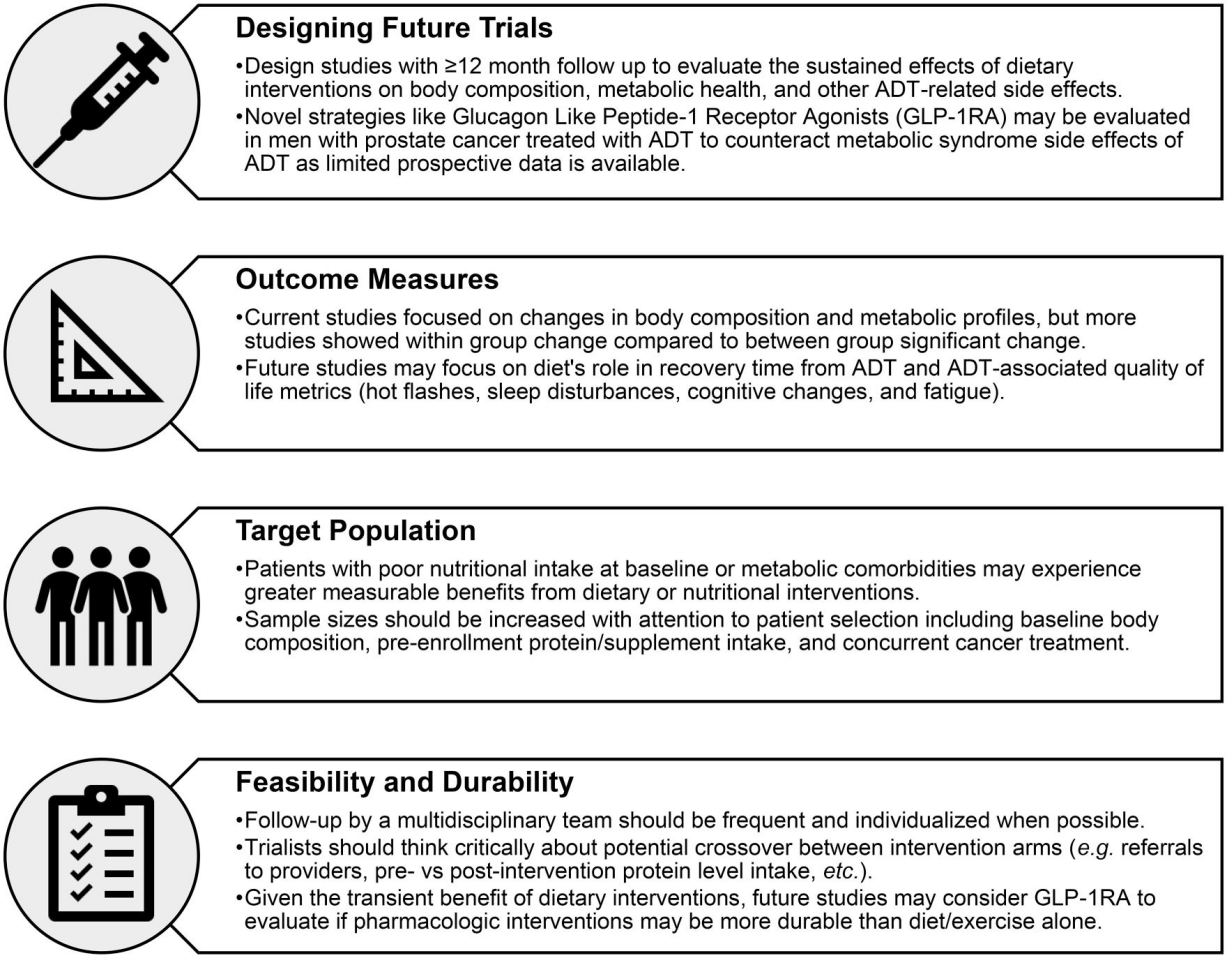
Future study design considerations and recommendations to evaluate diet and nutrition supplement interventions for men with prostate cancer treated with androgen deprivation therapy.

ADT may negatively impact musculoskeletal health and should be a focus of clinicians who recommend ADT for their patients (Figure 3). One tested strategy to mitigate this has been to leverage interventions aimed at increasing lean muscle mass and decreasing fat mass. In this review, there were trends towards increased lean mass across 3 RCTs ranging in duration from 12-week to 12-month interventions, although not usually statistically significant nor durable in long term benefit.^36^^,^^42^^,^^47^ These trials call into question the optimal duration of nutrition interventions as it relates to improving long term endpoints like lean muscle mass for men on ADT (Figure 4).

In the included studies, both creatine and whey protein interventions were not identified to consistently enhance muscle mass.^42^^,^^47^^,^^48^ However, two studies noted that their participants already consumed sufficient protein^48^ and had a more favorable metabolic profile^41^ at baseline, thus potentially explaining the lack of a significant effect. This suggests that further nutrition research is needed on the effects of supplementation among populations who do not regularly consume high levels of protein.^48^ Future studies may opt to stratify their population by baseline protein intake and overall health status to distinguish the effect of protein supplementation by baseline characteristics, or individualize protein intake to ensure each patient is receiving an appropriate dose according to their baseline levels (Figure 4).

Prior studies of creatine supplementation have suggested a 1-2 kg increase in lean muscle mass with creatine supplementation with or without exercise.^56^^,^^57^ However, an important distinction to make is that these studies have generally been in a eugonadal population. Thus, in the setting of ADT and without anabolic hormones to promote muscle growth, it is possible that creatine does not enhance muscle mass increases compared to exercise alone. Therefore, alternative strategies beyond creatine as supplementation may be explored if the desired outcome is increasing lean muscle mass. Nonetheless, it may be important to ascertain the optimal endpoint for similar creatine supplement trials moving forward (ie, physiologic endpoints like lean muscle mass versus behavioral endpoints like quality of life).

### Quality of life

Engagement in structured exercise programs also seems to affect improvements in quality of life and functional capacity. In a study by Focht et al., higher self-efficacy was associated with improved mobility performance and greater exercise participation.^37^ This relationship suggests a positive feedback loop where the ability to perform exercise interventions enhances confidence, resulting in sustained participation. These findings highlight the impactful role that personalized interventions play in not only improving self-efficacy, physical function, and mobility performance, but also physiological readiness, social support, and behavioral self-regulation to maximize adherence and functional improvement.

While sexual health is often a critical point for quality of life for men with prostate cancer on ADT, there were relatively fewer studies that focused on this outcome. Specifically, the relative time to recovery of sexual side effects may be worth evaluating in the future with diet and exercise interventions (Figure 4). This may be an important and timely area of future investigation given recent studies have highlighted that many men with prostate cancer do not feel adequately prepared by their providers on managing sexual-related side effects.^58^ Thus understanding how diet or supplement interventions may potentially impact sexual health in high-quality RCTs may be warranted.

### Feasibility and safety

Overall, the dietary interventions in this systematic review were considered safe without any severe toxicities recorded across all trials. Several interventions demonstrated feasibility and provided rationale for future survivorship studies: Mediterranean-diet and HIIT program,^36^ home-based interventions involving patient partners/caregivers,^44^ individualized counseling with multidisciplinary specialists to support survivorship,^41^ and a nurse-led exercise curriculum.^40^ A consistent theme was thus the importance of integrating a multidisciplinary care team and social support when conducting successful RCTs on nutrition in prostate cancer. However, given the potential burden this may put on the healthcare system, it may be possible that real world feasibility outside of a clinical trial environment could be limited.

There were no included studies that evaluated the important financial aspects of these interventions. There may be patients who could benefit from these interventions but face socioeconomic barriers that limit their ability to incorporate these dietary and lifestyle changes. This points to a larger public health issue that potentially limits the feasibility of dietary interventions in prostate cancer. A prior review on the barriers and facilitators of adopting the Mediterranean diet affirms that finances can prevent patients from adhering to recommended diet interventions.^59^ However, the authors mentioned that some healthy food options, like legumes, can be relatively affordable, suggesting that the Mediterranean diet may be adapted for individuals from lower-income settings. While there were mild GI and musculoskeletal side effects reported in some of the studies that are important to consider, no major adverse events were reported. This is helpful for clinicians to guide patients who are interested in these types of supplemental interventions while on treatment with ADT.

Multiple studies excluded patients with bone metastases, but one study recommended that clinicians prescribe a well-designed resistance exercise program to all patients beginning ADT treatment, including those with bone metastases who are not at high risk for pathological fractures.^48^ They advised that this exercise prescription should begin with supervision by an experienced therapist. Further investigation into the effects of nutrition and exercise interventions on patients with high-risk bone metastases is warranted.

### Limitations

There are several limitations to this current review. All studies had sample sizes under 100, which impacts the power of the results. The short-term follow-ups limit our ability to evaluate intervention durability. As mentioned previously, there were adherence concerns in some studies, which may bias the results. Fourteen interventions combined diet and nutrition components with exercise (Figure 2), limiting our ability to isolate the effects of diet. Interventions involving exercise are also difficult to blind, which may lead to biased results. Other limitations, like selective reporting, publication bias, and the inability to meta-analyze introduce further bias to the included studies. These limitations, as noted in both the ROB2 and GRADE appraisals of the studies, impacted the generalizability of these results. Also, given our objective was to capture modern ADT practices within the past decade, it is possible RCTs published over 10 years ago may have been excluded.

Given the variety of interventions and outcomes assessed in each study, there is an inherent limitation in the generalizability of the data. However, the GRADE table does appraise each type of intervention (diet and supplements) according to the most pertinent clinical outcomes. The lack of direct comparisons between the diets included in this review limits the ability to make strong clinical recommendations but emphasizes the need for future clinical trials that can make this comparison. Moreover, diet-related clinical trials are inherently challenging because patients are often aware of their assigned intervention, introducing bias.

#### Clinical implications

This review highlights that nutritional and educational programs are feasible when implemented in multidisciplinary settings. The benefits of participation were most apparent when lifestyle changes were maintained for at least 3 to 6 months and with frequent follow up by providers. Overall, interventions that target diet such as following a Mediterranean diet, structured exercise programs, and selective nutritional supplementation with vitamin D or protein may result in meaningful improvements to physical health and patient-reported outcomes, when compared to usual care. Possible recommendations for future RCTs (Figure 4) include optimizing follow-up time (eg, ≥12 months), primary outcome selection (eg, quality of life versus lab based metabolic profiling), population selection (eg, targeting populations with low baseline muscle mass or protein intake), and communication modalities (eg, multidisciplinary or community member communications).

### Future directions

Behavior change, especially related to diet and physical activity, can be difficult to sustain if the surrounding environment does not permit it. Participants in one study shared that they were interested in having their significant other involved in the education portion, particularly related to modifying dietary intake.^40^ Thus, there may be a benefit in creating a family-based nutrition and dietary modification curriculum tailored for men with prostate cancer undergoing ADT. One 6-month diet and physical activity home-based intervention involved the patient’s partner or caregiver and significantly reduced BMI without impacting fatigue, quality of life, or stress.^44^ This suggests that involving partners or caregivers in survivorship plans is a sustainable option for lifestyle interventions, as household support for dietary behavior change is crucial in maintaining long-term changes.

Although GLP-1RAs are primarily used for diabetes, and increasingly for obesity, emerging evidence also suggests they may play a role in prostate cancer prevention, treatment, and possibly survivorship.^60^^,^^61^ Despite including multiple key search terms for the GLP-1RA class of medications, the comprehensive evaluation of the RCT literature for men with prostate cancer treated with ADT did not yield any results. In fact, multiple RCTs in this systematic review specifically excluded patients who were taking weight modifying medications that generally include GLP-1RA.^17^^,^^37^^,^^39^^,^^44^^,^^47^^,^^50^^,^^51^^,^^61^ To our knowledge, there is only one active clinical trial (NCT06908694; GAIN PC CONTROL) investigating the safety and tolerability of semaglutide on men with prostate cancer.^62^ In this Phase 4 intervention trial, all men will be treated with ADT with or without an androgen receptor pathway inhibitor and the 1-year outcomes under investigation include weight, waist circumference, blood pressure, hemoglobin A1c, lipids, PSA, creatinine, and estimated glomerular filtration rate. This study is estimated to finish in July 2026, and the findings will likely impact clinical decision-making and could possibly improve short-term outcomes for men with prostate cancer. Since GLP-1RAs are effective for weight management, and weight gain is an adverse effect of ADT, studies should examine whether GLP-1RAs can impact long-term survivorship of patients with prostate cancer receiving ADT.^63^

Multidisciplinary team communications and community engagement has the potential of not only motivating participants to maintain dietary interventions, but it may also lead to more durable responses. Personalized and patient-tailored dietary intervention RCTs such as FRESH START underscored the importance of low-cost tailored educational materials that sustained behavioral change for at least 2 years for patients with breast and prostate cancer.^64^^,^^65^ Despite the changing landscape of patient communication, historic trials such as FRESH START, which used mail, demonstrated the importance of tailored patient engagement and highlighted the need for patient-focused communications for periods longer than 3 to 6 months. By doing this, future RCTs may be designed with sustainability in mind to best promote behavioral change when it comes to dietary modifications for patients with cancer.

## Conclusion

This systematic review identified 16 RCTs that evaluated nutritional interventions in the setting of men with prostate cancer treated with hormone therapy. While many studies yielded negative results for the primary outcome, the RCTs underscored the importance of designing trials with attention to baseline nutritional status, quality of life primary endpoints, multidisciplinary team support, and intervention duration. Given the promise of metabolic optimization with diet for men with prostate cancer receiving ADT, future studies may consider investigating longer term outcomes and metabolic regulating medications including incretin mimetics such as GLP-1RA.

## Supplementary Material

pkag014_Supplementary_Data

## Data Availability

Data is publicly available. Specific datasets will be shared upon application to the corresponding author.
