# Supplementary material for: Diet, nutrition, and hormone therapy for prostate cancer: a systematic review with implications for future interventions
Source: JNCI Cancer Spectr. 2026 Feb 17;10(2):pkag014. doi: 10.1093/jncics/pkag014 (PMC12972670; doi:10.1093/jncics/pkag014)
Supplement: pkag014_Supplementary_Data [file pkag014_supplementary_data.docx]

**Supplementary Material**

| Outcomes | Impact | No. participants (studies) | Evidence Certainty (GRADE) |
| --- | --- | --- | --- |
| **Diet Interventions** |  |  |  |
| **Weight/BMI** follow-up: 3-12 months | A mobile-based health coaching program had a reduction in body weight (F=10.71) and BMI (F=10.49) after 12 weeks (Lee *et al*) and a 6-month home-based diet and exercise counseling trial found a significant between-group reduction in weight and BMI (O’Neill *et al*). No significant difference in BMI was detected after a 12-month multidisciplinary clinic (Pollock *et al*). | 188 (3 RCTs) | ⨁⨁⨁◯ Moderate |
| **Lean Mass** (DEXA, BodPod)  follow-up: 3-12 months | A low-carbohydrate diet (Freedland *et al*) and Mediterranean diet intervention (Baguley *et al,* 2021) reduced lean body mass. A 6-month home-based diet and exercise counseling trial (O’Neill *et al*) and a Mediterranean diet study reported no change (Baguley *et al*, 2022). | 180 (4 RCTs) | ⨁⨁⨁◯ Moderate |
| **Overall Quality of Life** (FACT-P, PHQ-9, FACIT-G, Expanded Prostate Cancer Index Composite Short Form, Medical Outcomes Study 36-item Short Form Health Survey)  follow-up: 3-12 months | A home-based diet intervention (O’Neill *et al*), a group dietary counseling intervention (Focht *et al*, 2019), two Mediterranean diet intervention studies (Baguley *et al*, 2022 and Baguley *et al,* 2021), and a mobile diet and exercise intervention (Lee *et al*) resulted in an improvement in Quality of Life (QoL). There was no significant difference in QoL in a multidisciplinary clinic intervention (Pollock *et al*) and nutrition curriculum intervention (Myers *et al*). | 304 (7 RCTs) | ⨁⨁⨁◯ Moderate |
| **Fatigue (Quality of Life)**  (Fatigue Severity Scale, Lee Fatigue Scale, FACIT-F)  follow-up: 3-12 months | Mediterranean diet with exercise significantly improved cancer-related fatigue in two studies (Baguley *et al*, 2021 and Baguley *et al* 2022), improved functional well-being in a home-based diet counseling intervention (O’Neill *et al*), but found no difference in fatigue in one multidisciplinary clinic intervention (Pollock *et al*). | 188 (4 RCTs) | ⨁⨁⨁◯ Moderate |
| **LDL/HDL** (Blood lipid profile)  follow-up: 3-12 months | A low-carbohydrate diet resulted in sustained improvements in HDL at 6 months (Freedland *et al*). One nutrition curriculum/exercise intervention found no between-group difference in LDL (Myers *et al*), a counseling-based diet intervention had no difference (Gilbert *et al*), and a multidisciplinary clinic had trends toward improvements but no significant difference (Pollock *et al*). | 176 (4 RCTs) | ⨁⨁◯◯ Low |
| **Vasomotor Symptoms/Hot Flashes (Quality of Life)**  **(**Hot Flash Related Daily Interference Scale)  follow-up: 12 months | A diet and multidisciplinary clinic intervention had no significant improvement in hot flash symptoms (Pollock *et al*). | 48 (1 RCT) | ⨁⨁◯◯ Low |
| **HbA1c/Insulin Resistance** (Homeostatic model assessment)  follow-up: 6-12 months | A low-carbohydrate diet and walking intervention improved insulin resistance and HbA1c at 3 months but not at 6 months (Freedland *et al*) and a secondary analysis of the same study found that it had no effect on insulin resistance in serum (Chi *et al*). A multidisciplinary clinic intervention (Pollock *et al*) and nutritional curriculum (Myers *et al*) trended toward improvement in overall metabolic profile, but results were not statistically significant. | 155 (4 RCTs) | ⨁◯◯◯ Very low |
| **Supplement Interventions** |  |  |  |
| **Weight/BMI** follow-up: 3-12 months | A 12-month exercise and whey protein supplement intervention showed no significant difference in BMI (Dalla Via *et al*). | 70 (1 RCT) | ⨁⨁⨁◯ Moderate |
| **Lean Mass** assessed with: DEXA, BodPod follow-up: 3-12 months | A per protocol analysis of a whey protein intervention showed improved lean mass (Dalla Via *et al*). A creatine intervention trended towards increase in lean mass (Fairman *et al*). An exercise and protein supplement (Houben *et al*) and a Vitamin D intervention (Inglis *et al*) had no effect on lean mass. | 255 (4 RCTs) | ⨁⨁⨁◯ Moderate |

**Table S1.** Grading of Recommendations Assessment, Development, and Evaluation (GRADE) Table for Dietary and Nutritional Supplement Randomized Clinical Trial Interventions. GRADE appraisals are a compost score based on risk of bias, inconsistency, indirectness, imprecision, and publication bias.
